# Curcumin improves insulin sensitivity in high-fat diet-fed mice through gut microbiota

**DOI:** 10.1186/s12986-022-00712-1

**Published:** 2022-11-08

**Authors:** Yue Zhong, Yang Xiao, Jing Gao, Zhaozheng Zheng, Ziheng Zhang, Lu Yao, Dongmin Li

**Affiliations:** 1 Department of Biochemistry and Molecular Biology, School of Basic Medical Sciences, Xi’an Jiaotong University Health Science Center, Western Yanta Road 76, Xi’an, 710061 Shanxi Province People’s Republic of China; 2 College of Management, Jilin Medical University, No.5 Jilin Street, Jilin, 132013 Jilin Province People’s Republic of China; 3 College of Pharmacy, Jilin Medical University, No.5 Jilin Street, Jilin, 132013 Jilin Province People’s Republic of China; 4 College of Basic Medical Sciences, Jilin Medical University, No.5 Jilin Street, Jilin, 132013 Jilin Province People’s Republic of China; 5 Experimental Center of Research on Prevention and Treatment of Chronic Diseases, Jilin Medical University, No.5 Jilin Street, Jilin, 132013 Jilin Province People’s Republic of China

**Keywords:** Insulin sensitivity, Gut microbiota, Curcumin, Glucose and lipid metabolism, FGF15

## Abstract

**Background:**

Insulin resistance precedes metabolic syndrome which increases the risk of type 2 diabetes and cardiovascular disease. However, there is a lack of safe and long-lasting methods for the prevention and treatment of insulin resistance. Gut microbiota dysbiosis can lead to insulin resistance and associated glucose and lipid metabolic dysfunction. Thus, the role of gut microbiota in metabolic diseases has garnered growing interest. Curcumin, the active ingredient of tropical plant *Curcuma longa*, has excellent prospects for the prevention and treatment of metabolic diseases. However, due to the extremely low bioavailability of curcumin, the mechanisms by which curcumin increases insulin sensitivity remains to be elucidated. This study aimed to elucidate the role of gut microbiota in mediating the effects of curcumin on improving insulin sensitivity in high-fat diet (HFD)-fed mice.

**Methods:**

Glucose, insulin, and pyruvate tolerance were tested and hepatic triglycerides (TGs) content was measured in HFD-fed mice treated with curcumin (100 mg kg^−1^ d^−1^, p.o.) or vehicle for 4 weeks and aforementioned mice after gut microbiota depletion via antibiotic treatment for 4 weeks. Fecal microbiota transplantation (FMT) was conducted in endogenous gut microbiota-depleted HFD-fed mice. Glucose and lipid metabolic phenotypes were also measured in recipient mice colonized microbiota from vehicle- or curcumin-treated HFD-fed mice. The mechanisms underlying the effects of curcumin on increasing insulin sensitivity were testified by Western blotting, real-time quantitative polymerase chain reaction and enzyme-linked immunosorbent assay (ELISA).

**Results:**

Curcumin ameliorated HFD-induced glucose intolerance, insulin resistance, pyruvate intolerance, and hepatic TGs accumulation, while these effects were mediated by gut microbiota. Curcumin induced insulin-stimulated Akt phosphorylation levels in insulin-regulated peripheral tissues. The inhibitory effects of curcumin on the expressions of genes involved in hepatic gluconeogenesis and de novo lipogenesis were dependent on gut microbiota. Meanwhile, curcumin upregulated the expression of fibroblast growth factor 15 (FGF15) through gut microbiota.

**Conclusions:**

The effects of curcumin on promoting insulin sensitivity were dependent on gut microbiota in HFD-fed mice. Moreover, curcumin at least partly exerted its effects on increasing insulin sensitivity via FGF15 upregulation. This study provided new ideas on nutritional manipulations of gut microbiota for the treatment of metabolic diseases.

**Supplementary Information:**

The online version contains supplementary material available at 10.1186/s12986-022-00712-1.

## Background

The increasing prevalence of sedentary lifestyles and high-fat, high-sugar diets have contributed to a growing number of adults suffering from metabolic syndrome. Metabolic syndrome refers to a group of clinical syndromes characterized by abdominal obesity, dyslipidemia, hypertension, and insulin resistance that also significantly increases the risk of cardiovascular disease and type 2 diabetes [1]. According to estimates, one in four adults around the world will be diagnosed with metabolic syndrome [2]. Insulin resistance resulting from environmental factors (e.g. obesity, lack of exercise, etc.) is an important cause of metabolic syndrome [3]. Furthermore, insulin resistance also precedes the onset of type 2 diabetes [4]. Thus, it is evident that improving insulin sensitivity has significant implications for the prevention and treatment of metabolic syndrome and type 2 diabetes. Unfortunately, there is currently a lack of adequate methods and approaches to ensure the safe and long-lasting alleviation of insulin resistance.

Accumulating studies have found that gut microbiota are involved in the regulation of glucose and lipid metabolism [5]. Germ-free mice fed a HFD did not develop insulin resistance, however, germ-free mice who received fecal microbiota transplantation (FMT) from normal mice did develop insulin resistance and glucose intolerance induced by HFD [6, 7]. Clinical trials have also demonstrated that gut microbiota dysbiosis is an important cause of insulin resistance, and FMT from lean donors improved the insulin resistance of patients with metabolic syndrome [8, 9]. However, the mechanisms underlying the regulation of insulin sensitivity by the gut microbiota remain poorly understood. FGF15 is a metabolic hormone synthesized and secreted by ileal enterocytes. As a member of the FGF family, this gut-derived hormone shares similar physiological functions as FGF21 and FGF1 in enhancing insulin sensitivity and mitigating glucose and lipid metabolic disorders (GLMDs) [10, 11]. Moreover, gut microbiota can regulate farnesoid X receptor (FXR) activity through their metabolites, which in turn can affect FGF15 expression, suggesting that FGF15 is highly likely to be involved in the regulation of host insulin sensitivity by gut microbiota [12, 13]. Given that the basic physiological function of gut microbiota is to assist the host in digesting complex foods, it follows that diet can affect the composition of gut microbiota, which implies that insulin resistance can be alleviated through the consumption of specific foods that can mitigate gut microbiota dysbiosis, thereby achieving the prevention and treatment of insulin resistance-induced metabolic syndrome and type 2 diabetes.

Curcumin is a polyphenolic compound extracted from tropical plant *Curcuma longa*, and as a phytochemical derived from a medicinal and edible food, it not only has an extremely low level of toxicity but also has been used in traditional medicine to improve metabolic diseases. Moreover, curcumin has been shown to improve metabolic syndrome in basic research, and its potential efficacy in improving diabetes has also been demonstrated in clinical trials. Therefore, curcumin has excellent prospects for clinical application in the prevention and treatment of insulin resistance [14–16]. However, curcumin has an extremely low bioavailability, while oral administration is subjected to the first pass effect, which results in an extremely low concentration in circulation. The poor oral bioavailability of curcumin infer that its beneficial effects are most likely exerted through gut microbiota [17–19]. Thus, the mechanisms of curcumin in preventing and treating insulin resistance remains to be elucidated. In a recent study by our research team, gut microbiota were shown to mediate the enhancement of uncoupling protein 1-dependent thermogenesis by curcumin in HFD-fed mice, while curcumin increased energy consumption by improving HFD-induced gut microbiota dysbiosis and hence ameliorating HFD-induced obesity in mice [20]. Accordingly, we speculated that curcumin may exert its protective effects against insulin resistance via gut microbiota modulation. We aim to elucidate the role of gut microbiota in the effects of curcumin on ameliorating insulin resistance in HFD-fed mice. We believe this knowledge would provide a theoretical basis for the clinical application of curcumin in the prevention and treatment of metabolic syndrome and type 2 diabetes, as well as new ideas for the treatment of metabolic diseases.

## Methods

### Animals and experimental design

Specific pathogen-free (SPF) 8-week-old male C57BL/6J mice purchased from Charles River Laboratories (Beijing, China) were housed and maintained under a 12 h light/dark photoperiod at a constant temperature (23 °C) with unlimited availability of water and food. Mice were fed a HFD (D12492; Research Diets, New Brunswick, NJ, USA). Mice were grouped randomly into two groups. Curcumin dissolved in 0.5% carboxymethylcellulose was administered to mice daily by intragastric gavage since mice were fed with HFD; 0.5% carboxymethylcellulose sodium was administered to the vehicle group as controls. Based on our previous research, we chose 100 mg per kg bodyweight as the gavage dose of curcumin [20]. HFD-fed mice after 4 weeks’ gavage experiment were used to examine glucose and lipid metabolic phenotypes. To deplete endogenous gut microbiota from C57BL/6J mice after 4 weeks of treatment with curcumin or vehicle during HFD feeding, antibiotics were administered via drinking water, and replaced with freshly prepared antibiotics every second day for 4 weeks. The antibiotics regimen was neomycin (100 μg mL^−1^), streptomycin (50 μg mL^−1^), penicillin (100 U mL^−1^), vancomycin (50 μg mL^−1^), metronidazole (100 μg mL^−1^), bacitracin (1 mg mL^−1^), ciprofloxacin (125 μg mL^−1^), ceftazidime (100 μg mL^−1^), and gentamycin (170 μg mL^−1^). Meanwhile, antibiotics-treated mice were administered curcumin or vehicle, as appropriate, every day. After depleted endogenous gut microbiota, curcumin- and vehicle-treated HFD-fed mice were used to examine glucose and lipid metabolic phenotypes. SPF 4-week-old male C57BL/6J mice were used to generate endogenous gut microbiota-depleted recipient mice. FMT was undertaken based on an established protocol [9]. Briefly, 500 mg of fresh feces from curcumin-treated or vehicle-treated HFD-fed mice was resuspended in 5 mL of sterile reduced phosphate-buffered saline. The suspension was allowed to settle by gravity for 5 min, after which a 200 μL aliquot of supernatant was administrated to recipient mice by intragastric gavage. All recipient mice were fed with HFD, and FMT was carried out daily. Fresh transplant materials were prepared on the same day of transplantation, < 10 min before gavage, to prevent changes in bacterial composition. After transplanted with microbiota from curcumin- or vehicle-treated HFD-fed mice for 4 weeks, recipient mice were used to examine glucose and lipid metabolic phenotypes. For all experiments in this study, male and age-matched mice using the corresponding treatment were used. All mice went through a 6 h fast under deep isoflurane induced anesthesia before blood was collected from the inferior vena cava, and tissues were harvested for further analyses.

### Reagents

Curcumin (purity ≥ 95.0%) was purchased from Shanghai Yuanye Biotech (Shanghai, China). Carboxymethylcellulose sodium was obtained from Sigma-Aldrich (Saint Louis, MO, USA). Neomycin, streptomycin, penicillin, vancomycin, metronidazole, bacitracin, ciprofloxacin, ceftazidime and gentamycin were purchased from Sangon Biotech (Shanghai, China). TRIzol® Reagent was from Invitrogen (Carlsbad, CA, USA). High-Capacity cDNA Reverse- Transcription Kits were purchased from Applied Biosystems (Foster City, CA, USA). SYBR Green PCR Master Mix was obtained from Promega (Fitchburg, MI, USA). Proteinase inhibitor cocktail and phosphorylase inhibitor were purchased from Roche (Basel, Switzerland). Bicinchoninic acid (BCA) Protein Assay Kits were purchased from Genstar Technologies (Beijing, China). Antibodies against Akt (9272), pAkt (9271) were purchased from Cell Signaling Technology (Danvers, MA, USA). The antibody against FGF15 (ab229630) was obtained from Abcam. The antibody against Tubulin (CW0098) was from Cwbiotech (Beijing, China).

### Glucose, insulin and pyruvate tolerance test

For glucose and pyruvate tolerance test experiments, mice fasted for 16 h were injected intraperitoneally with D-glucose or pyruvate (2 g/kg). For insulin tolerance test experiment, mice fasted for 6 h were injected intraperitoneally with human insulin (Sigma) (0.5 U/kg). Blood glucose levels were measured from the tail vein at indicated times using a glucometer (One Touch Ultra; LifeScan Inc., Milpitas, CA).

### Biochemical analyses

Hepatic TGs levels were determined using a commercial kit following the manufacturer’s protocol (Applygen Technologies, Inc, Beijing, China). Serum FGF15 concentrations were measured by ELISA following the manufacturer’s protocol (CUSABIO, Wuhan, China).

### Quantitative real-time PCR

Total RNA was extracted from tissues with TRIzol Reagent according to manufacturer instructions. For quantitative real-time PCR analysis, 2 μg of total RNA was reverse-transcribed using the High-Capacity cDNA Reverse-Transcription kit. SYBR Green reactions using SYBR Green PCR Master Mix were assembled along with 500 nM primers according to manufacturer instructions and undertaken with a C1000 Thermal Cycler CFX96 Real-Time system (Bio-Rad Laboratories, Hercules, CA, USA) or an ABI 7500-Fast Real-Time PCR system (Applied Biosystems). Relative expression of mRNAs was determined after normalization to ribosomal protein S18 (Rps18). Primer sequences are shown in Table 1.

**Table 1:** Primers sequences used for real-time quantitative PCR gene expression analysis

Gene	Forward primer (5′–3′)	Reverse primer (5′–3′)
*Pck1*	CTGCATAACGGTCTGGACTTC	CAGCAACTGCCCGTACTCC
*G6pc*	CGACTCGCTATCTCCAAGTGA	GTTGAACCAGTCTCCGACCA
*Srebf1*	TGCGGCTGTTGTCTACCATA	TGCTGGAGCTGACAGAGAAA
*Fasn*	TATCAAGGAGGCCCATTTTGC	TGTTTCCACTTCTAAACCATGCT
*Acaca*	ATGGGCGGAATGGTCTCTTTC	TGGGGACCTTGTCTTCATCAT
*Scd1*	TTCTTGCGATACACTCTGGTGC	CGGGATTGAATGTTCTTGTCGT
*Fgf15*	ATGGCGAGAAAGTGGAACGG	CTGACACAGACTGGGATTGCT
*Rps18*	TTCCAGCACATTTTGCGAGTA	CACGCCCTTAATGGCAGTGAT

### Protein extraction and western blotting

Tissue lysates were prepared using RIPA buffer supplemented with a proteinase inhibitor cocktail, phenylmethylsulfonyl fluoride and phosphorylase inhibitor. Protein concentrations were determined using the BCA Protein Assay Kit. Protein from the indicated samples was separated by sodium dodecyl sulfate–polyacrylamide gel electrophoresis and transferred onto polyvinylidene difluoride membranes. The latter were incubated with the respective polyclonal antibodies. After washing, horseradish peroxidase-conjugated anti-rabbit immunoglobulin (Ig)G and anti-mouse IgG were used as the secondary antibody at 1: 5 000 dilution. Relative protein images were determined using horseradish peroxidase-conjugated secondary antibodies and electrochemiluminescence substrates. The intensities of the immunoreactive bands were quantified by densitometry using Image Lab (Bio-Rad Laboratories).

### Statistical analyses

Numerical data are presented as the mean ± SEM. Statistical analyses were carried out using SPSS 20 (IBM, Armonk, NY, USA). *P* < 0.05 was considered significant and determined by two-tailed Student’s t tests (for comparison of two experimental conditions) or ANOVA (for comparison of more than two experimental conditions) followed by Bonferroni’s test. The number of animals used for each experiment is indicated in the respective figure legends.

## Results

### Curcumin ameliorated HFD-induced GLMDs

**Fig. 1:**
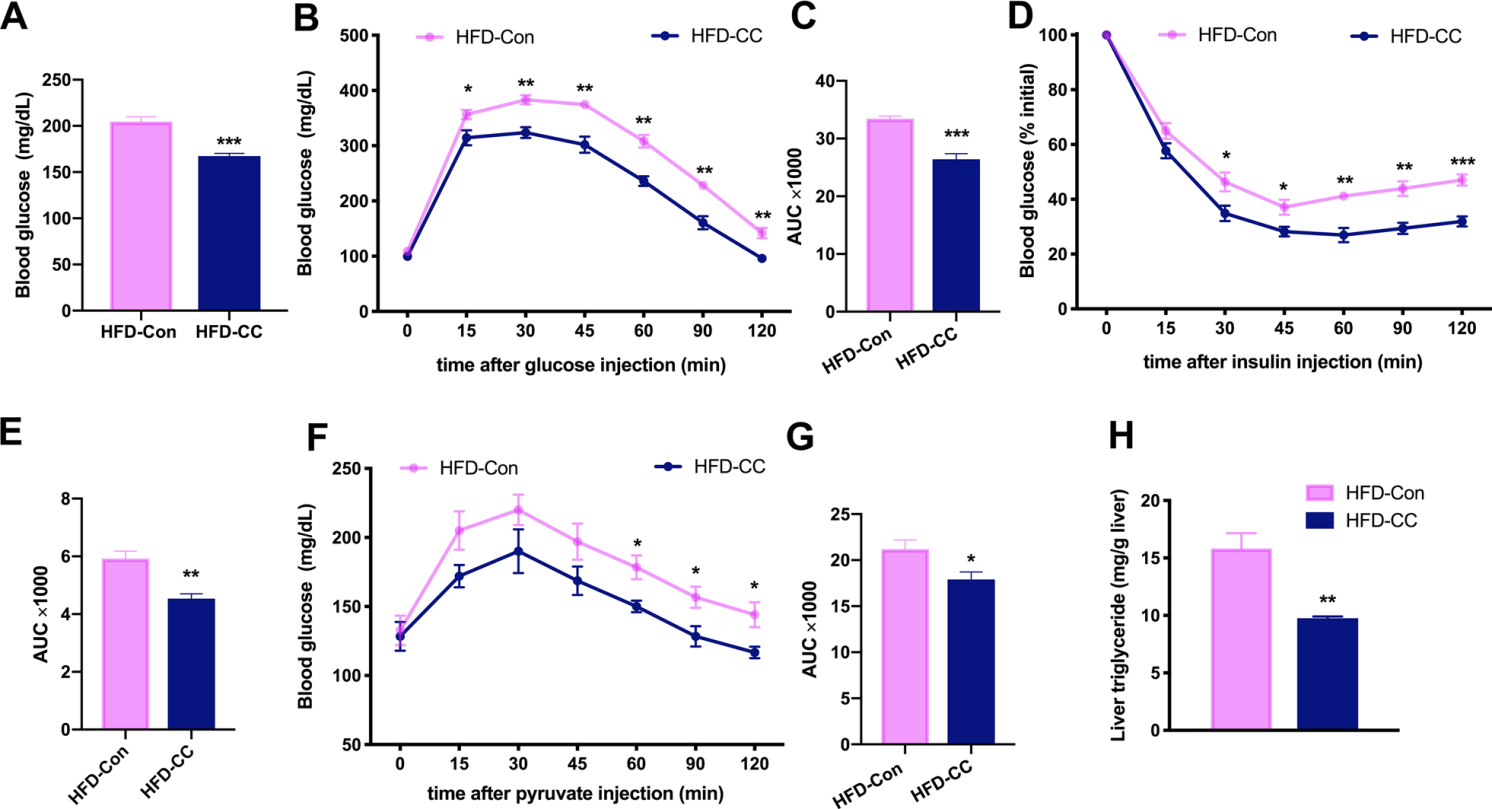
Effects of curcumin on glucose and lipid metabolism in HFD-fed mice. **A** Serum glucose levels in 6-h-fasted HFD mice treated with curcumin or vehicle for 4 weeks (*n* = 8/group). **B**–**G** Blood glucose concentrations during an intraperitoneal GTT (**B**), ITT (**D**) and PTT (**F**) performed in mice as in (**A**). Area under the curve (AUC) of GTT (**C**), ITT (**E**) and PTT (**G**) (n = 6/group). **H** Hepatic TGs levels of mice as in **A** (n = 8/group). Numerical data are shown as the mean ± SEM. *P < 0.05, **P < 0.01, ***P < 0.001

8-week-old male C57BL/6J mice fed with HFD were randomly divided into two groups. One group was administered curcumin via gavage, while the other group was administered corresponding control solution. Although curcumin can reduce HFD-induced body weight gain, there was no significant difference in the body weight when curcumin- and vehicle-treated mice were fed with HFD for 4 weeks (Additional file 1: Fig S1). Fasting blood glucose was measured before a significant difference in body weight was detected between the two groups, and the results revealed that curcumin mitigated the HFD-induced increase in fasting blood glucose (Fig. 1A). Moreover, glucose and insulin tolerance tests (GTT and ITT) showed that curcumin improved HFD-induced glucose intolerance and insulin resistance, respectively (Fig. 1B–E). The pyruvate tolerance test (PTT) implied that curcumin inhibited the hepatic gluconeogenesis (Fig. 1F, G). 4 weeks of HFD was insufficient to cause obvious features of fatty liver in mice (data not shown), and there is no difference in the liver weight between the two groups (Additional file 1: Fig. S2). However, measurement of hepatic TGs content revealed that curcumin reduced the accumulation of TGs in the liver of HFD-fed mice (Fig. 1H). These findings suggested that curcumin could enhance the insulin sensitivity of HFD-fed mice, thereby ameliorating HFD-induced GLMDs.

### The beneficial effects of curcumin on improving HFD-induced GLMDs were dependent on gut microbiota

**Fig. 2:**
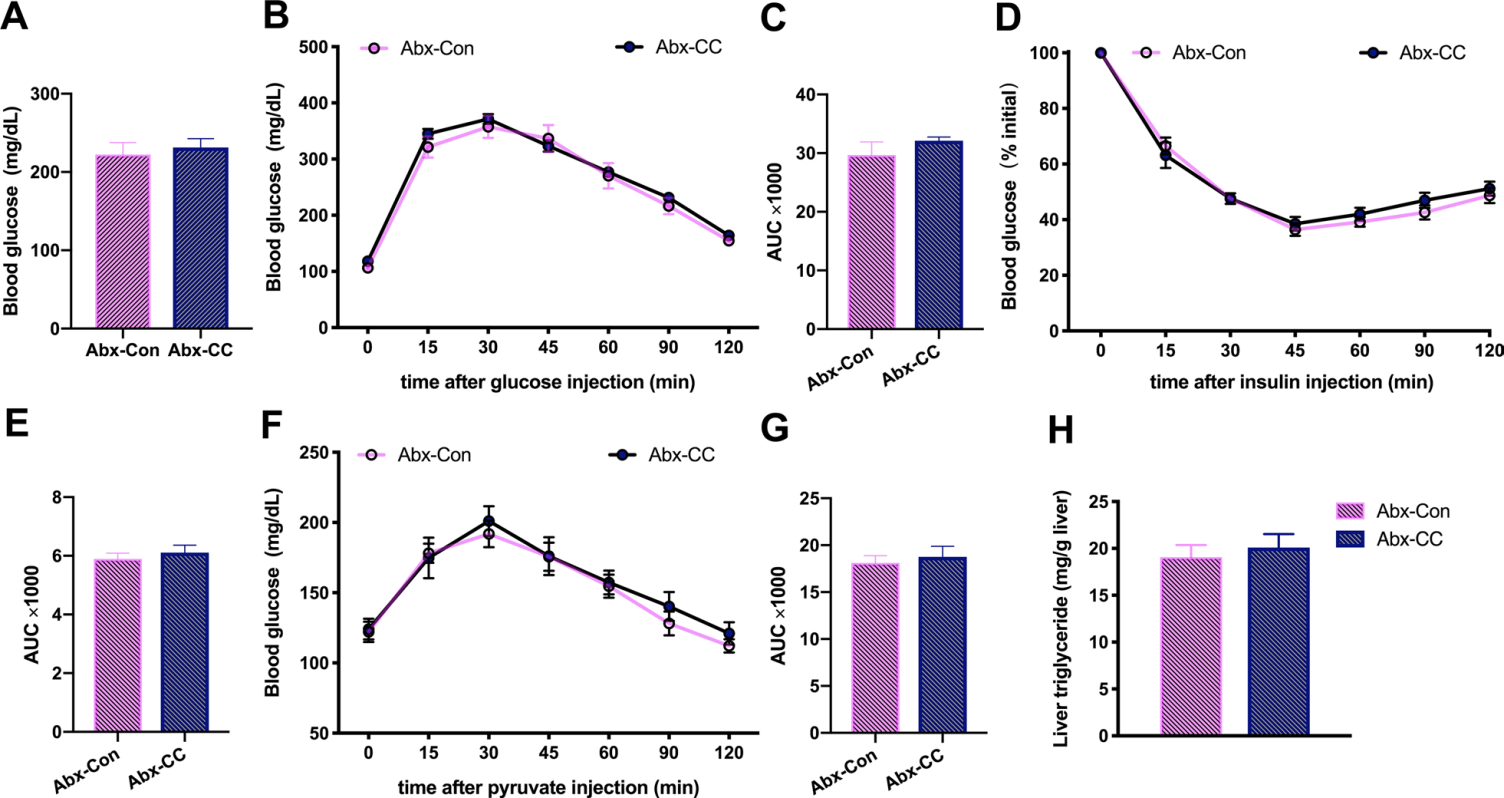
Effects of curcumin on glucose and lipid metabolism in HFD-fed mice after antibiotic treatment. **A** Serum glucose levels in 6-h-fasted vehicle- and curcumin-treated HFD-fed mice after antibiotic treatment (*n* = 8/group). **B**–**G** Blood glucose concentrations during an intraperitoneal GTT (**B**), ITT (**D**) and PTT (**F**) performed in mice as in **A**. Area under the curve (AUC) of GTT (**C**), ITT (**E**) and PTT (**G**) (n = 8/group). **H** Hepatic TGs levels of mice as in **A** (n = 8/group). Numerical data are shown as the mean ± SEM

Based on the poor oral bioavailability of curcumin and our previous research results, we speculated that curcumin may exert its beneficial effects on GLMDs via gut microbiota modulation [17, 18, 20]. To verify this hypothesis, a variety of broad-spectrum antibiotics were added to the drinking water of two groups of mice subjected to the gavage experiment. The mice were given free access to this drinking water for 4 weeks to deplete their endogenous gut microbiota. Curcumin- and vehicle-treated HFD-fed mice showed similar body weight gain during gut microbiota depletion (Additional file 1: Fig. S3). Subsequently, fasting blood glucose was measured in both groups of mice with depleted gut microbiota, and showed that the beneficial effects of curcumin on reducing HFD-induced hyperglycemia had disappeared (Fig. 2A). Furthermore, GTT and ITT revealed that both groups of mice with depleted gut microbiota exhibited similar levels of glucose tolerance and insulin sensitivity (Fig. 2B–E). Similarly, results of PTT showed that gut microbiota depletion eliminated the inhibitory effects of curcumin on gluconeogenesis (Fig. 2F, G). Furthermore, gut microbiota depletion reversed the protective effects of curcumin against the accumulation of hepatic TGs (Fig. 2H). These findings indicated that the amelioration of HFD-induced GLMDs induced by curcumin relied on gut microbiota.

### Curcumin-restructured fecal microbiota improved HFD-induced GLMDs

**Fig. 3:**
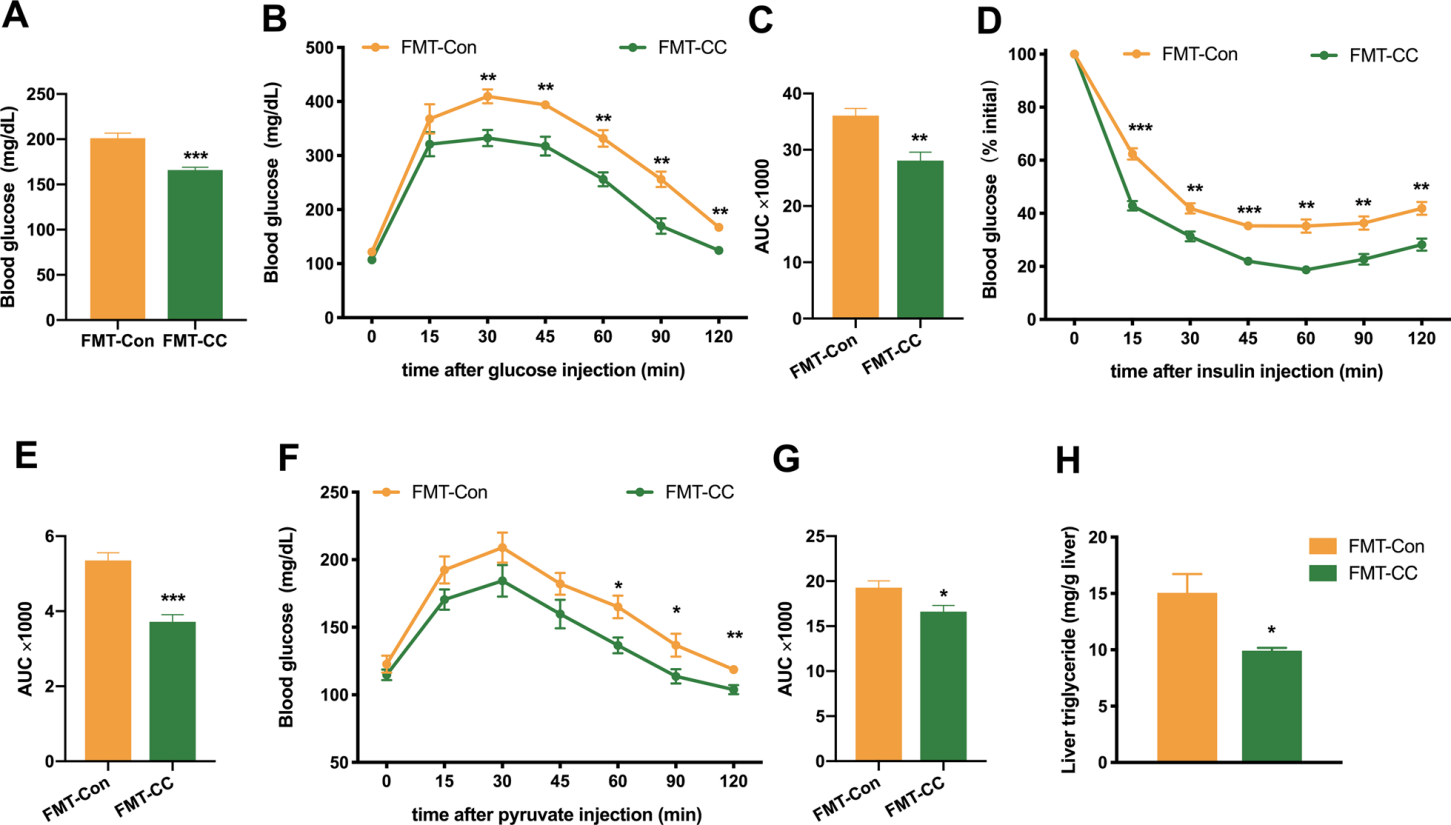
Effects of curcumin-restructured fecal microbiota on glucose and lipid metabolism in endogenous gut microbiota-depleted mice. **A** Serum glucose levels in 6-h-fasted endogenous gut microbiota-depleted mice colonized with the microbiota harvested from curcumin- and vehicle-treated HFD-fed mice during HFD feeding (*n* = 8/group). **B**–**G** Blood glucose concentrations during an intraperitoneal GTT (**B**), ITT (**D**) and PTT (**F**) performed in mice as in **A**. Area under the curve (AUC) of GTT (**C**), ITT (**E**) and PTT (**G**) (n = 8/group). **H** Hepatic TGs levels of mice as in **A** (n = 8/group). Numerical data are shown as the mean ± SEM. *P < 0.05, **P < 0.01, ***P < 0.001

The results of our previous studies demonstrated that curcumin could restructure gut microbiota community and improve HFD-induced gut microbiota dysbiosis [20]. In the current study, FMT was performed to further verify the hypothesis that curcumin exerts its protective effects against insulin resistance via gut microbiota modulation. Compared to curcumin treatment, curcumin-restructured gut microbiota displayed similar impact on HFD-induced body weight gain (Additional file 1: Fig. S4). Our findings revealed that the transplantation of fecal microbiota from curcumin-treated HFD-fed mice decreased the fasting blood glucose of recipient mice with depleted endogenous gut microbiota compared to that of the control group (Fig. 3A). Moreover, GTT and ITT showed that the curcumin-restructured fecal microbiota could improve HFD-induced glucose intolerance and insulin resistance (Fig. 3B–E). PTT implied that the curcumin-restructured fecal microbiota could inhibit hepatic gluconeogenesis (Fig. 3F, G). In addition, the fecal microbiota from curcumin-treated HFD-fed mice significantly mitigated the HFD-induced accumulation of hepatic TGs (Fig. 3H). Taken together, these results indicated that the curcumin-restructured fecal microbiota exhibited similar efficacy to curcumin in improving HFD-induced GLMDs, thus further demonstrating that the beneficial effects of curcumin were mediated by gut microbiota.

### Curcumin promoted insulin sensitivity through gut microbiota modulation in HFD-fed mice

**Fig. 4:**
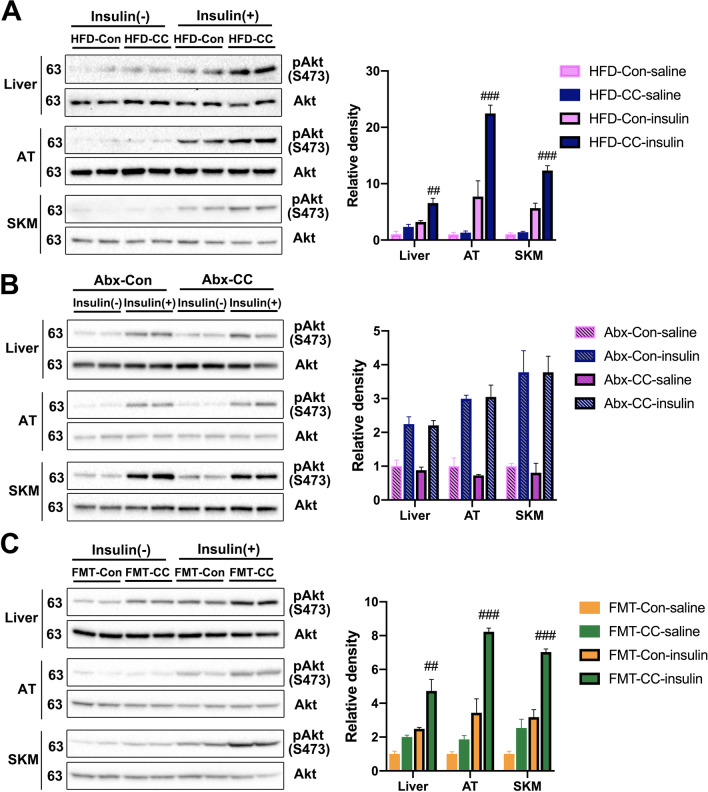
Curcumin improves HFD-induced insulin resistance through gut microbiota. **A**–**C** Western blotting analysis (left panel) of phosphorylated Akt and total Akt in liver, adipose tissue (AT) and skeletal muscle (SKM) extracts from curcumin- and vehicle-treated HFD-fed mice (**A**), vehicle- and curcumin-treated HFD-fed mice after antibiotic treatment (**B**) and endogenous gut microbiota-depleted mice colonized with the microbiota harvested from curcumin- and vehicle-treated HFD-fed mice (**C**) with or without insulin stimulation for 30 min. Right panel shows the result of densitometric analyses. Numerical data are shown as the mean ± SEM. ^##^*P* < 0.01, ^###^*P* < 0.001 for HFD-Con- insulin versus HFD-CC-insulin or FMT-Con-insulin versus FMT-CC-insulin

Based on the phenotypic analyses aforementioned, we concluded that curcumin might increase insulin sensitivity through gut microbiota modulation. To verify it, Akt phosphorylation levels were measured in the liver, adipose tissue, and skeletal muscle, which revealed that curcumin significantly upregulated insulin-stimulated Akt phosphorylation in these tissues (Fig. 4A). Conversely, gut microbiota depletion eliminated the effects of curcumin on upregulating insulin-stimulated Akt phosphorylation (Fig. 4B). Furthermore, measurements of FMT recipient mice showed that the curcumin-restructured fecal microbiota upregulated the insulin-stimulated increase in Akt phosphorylation (Fig. 4C). Therefore, these results demonstrated that curcumin could enhance insulin sensitivity in HFD-fed mice, and this effect required gut microbiota.

### Curcumin alleviated hepatic gluconeogenesis and de novo lipogenesis through gut microbiota modulation in HFD-fed mice

**Fig. 5:**
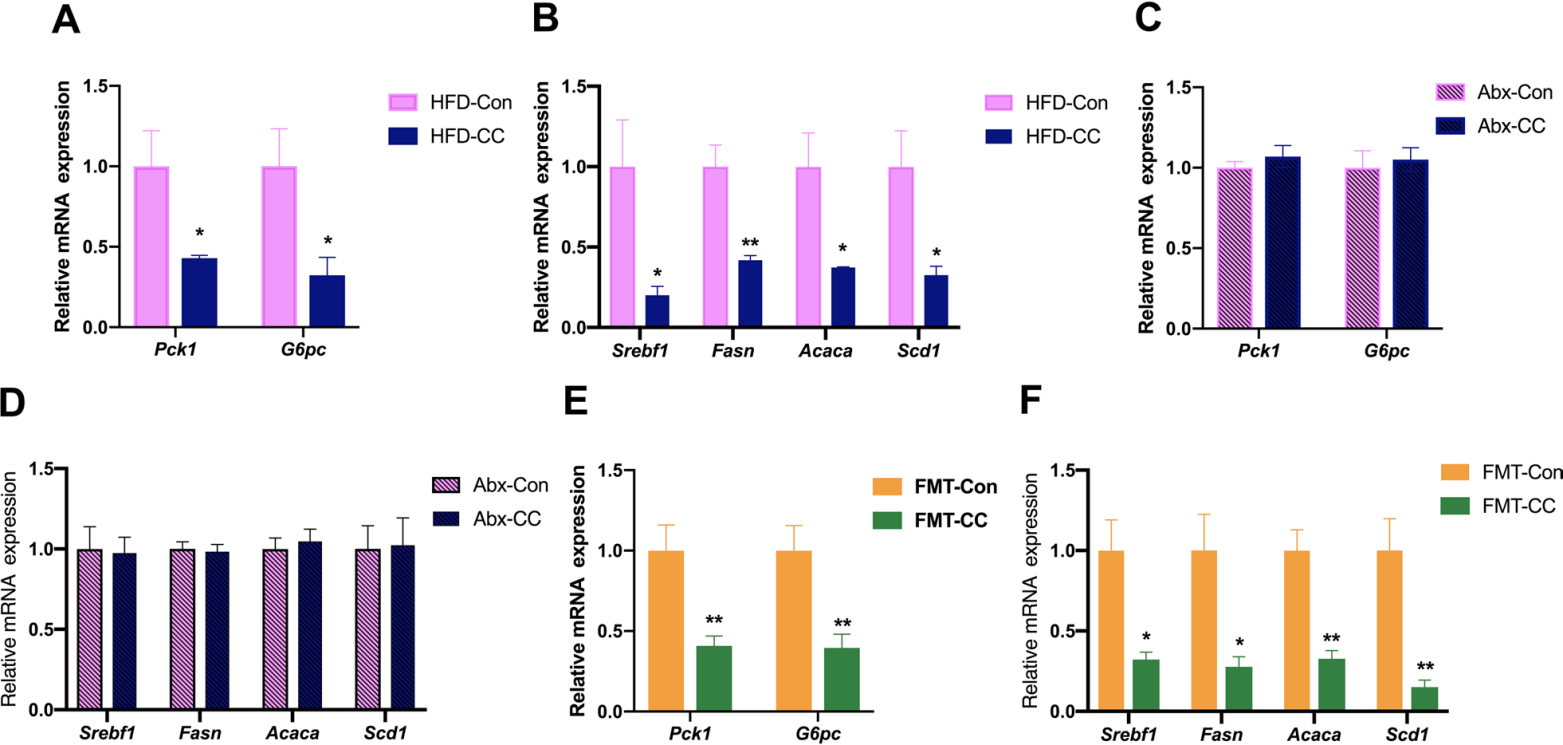
Curcumin regulates genes involved in hepatic gluconeogenesis and de novo lipogenesis through gut microbiota. **A** Real-time quantitative PCR analysis of *Pck1* and *G6pc* expression in the liver from HFD-fed mice treated with curcumin or vehicle for 4 weeks (n = 6/group). **B** Real-time quantitative PCR analysis of genes involved in DNL in the liver from mice in **A** (n = 6/group). **C**, **D** Real-time quantitative PCR analysis of *Pck1* and *G6pc* expression (**C**) and genes involved in DNL (**D**) in the liver from vehicle- and curcumin-treated HFD-fed mice after antibiotic treatment (n = 6/group). **E**, **F** Real-time quantitative PCR analysis of *Pck1* and *G6pc* expression (**E**) and genes involved in DNL (**F**) in the liver from endogenous gut microbiota-depleted mice colonized with the microbiota harvested from curcumin- and vehicle-treated HFD-fed mice (n = 6/group). Numerical data are shown as the mean ± SEM. *P < 0.05, **P < 0.01

Insulin resistance upregulates the expression of genes involved in the hepatic gluconeogenesis and de novo lipogenesis (DNL), which leads to the abnormal enhancement of the hepatic gluconeogenesis and DNL, thereby inducing hyperglycemia and hepatosteatosis [4, 21, 22]. Our findings implied that curcumin could reduce hepatic gluconeogenesis and DNL in HFD-fed mice. To verify it, we measured the mRNA expression levels of key gluconeogenic enzymes and found that curcumin reduced the expression of *Pck1* and *G6pc* (Fig. 5A). In addition, curcumin also significantly inhibited the expression of *Srebf1* and its target genes involved in hepatic DNL in HFD-fed mice (Fig. 5B). Moreover, gut microbiota depletion abolished the effects of curcumin on reducing the expression of genes involved in hepatic gluconeogenesis and DNL (Fig. 5C, D). Furthermore, curcumin-restructured fecal microbiota could mimic the beneficial effects of curcumin on regulating genes involved in hepatic gluconeogenesis and DNL (Fig. 5E, F). In conclusion, these results indicated that curcumin reduced hepatic gluconeogenesis and DNL in HFD-fed mice through gut microbiota modulation.

### Curcumin upregulated the expression of FGF15 through gut microbiota in HFD-fed mice

**Fig. 6:**
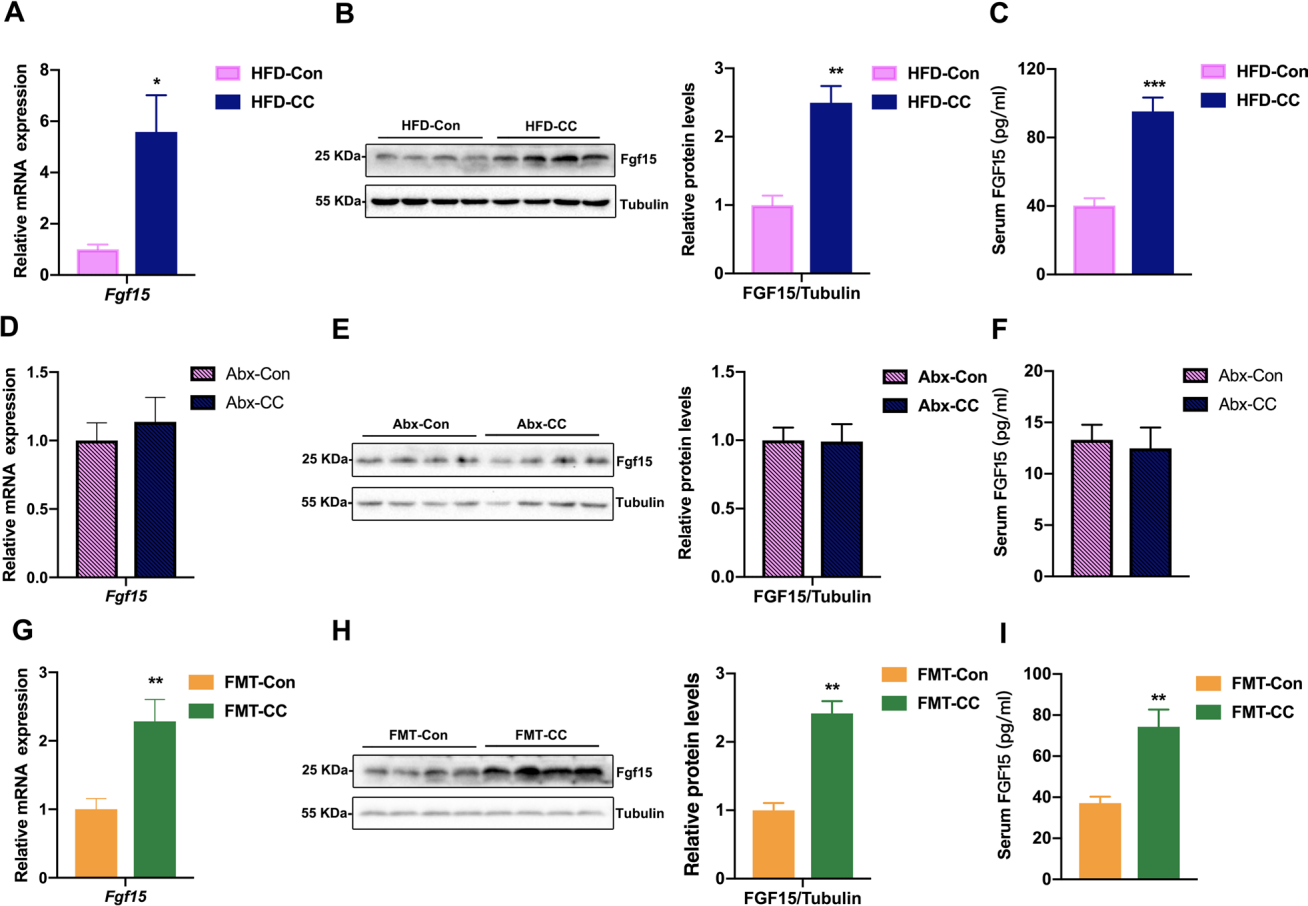
Gut microbiota mediate the effects of curcumin on regulating FGF15. **A** Real-time quantitative PCR analysis of *Fgf15* expression in the ileum of HFD mice treated with curcumin or vehicle for 4 weeks (n = 8/group). **B** Western blotting (left panel) and densitometric analyses (right panel) of FGF15 in the ileum from mice in **A**. **C** Serum FGF15 levels of mice in **A** (n = 8/group). **D** Real-time quantitative PCR analysis of *Fgf15* expression in the ileum of vehicle- and curcumin-treated HFD-fed mice after antibiotic treatment (n = 8/group). **E** Western blotting (left panel) and densitometric analyses (right panel) of FGF15 in the ileum from mice in **D**. **F** Serum FGF15 levels of mice in **D** (n = 8/group). **G** Real-time quantitative PCR analysis of *Fgf15* expression in the ileum of endogenous gut microbiota-depleted mice colonized with the microbiota harvested from curcumin- and vehicle-treated HFD-fed mice during HFD feeding (n = 8/group). **H** Western blotting (left panel) and densitometric analyses (right panel) of FGF15 in the ileum from mice in **G**. **I** Serum FGF15 levels of mice in **G** (n = 8/group). Numerical data are shown as the mean ± SEM. *P < 0.05, **P < 0.01, ***P < 0.001

FGF15, a gut-derived hormone, is regulated by gut microbiota and involved in the regulation of glucose and lipid metabolism, so it’s necessary to investigate whether curcumin had an impact on FGF15 expression [12]. Our findings indicated that curcumin upregulated FGF15 expression (Fig. 6A, B). Curcumin-treated HFD-fed mice also showed significantly elevated levels of serum FGF15 (Fig. 6C). Moreover, gut microbiota depletion abolished the upregulation effect of curcumin on the expression and serum level of FGF15 (Fig. 6D–F). Conversely, the fecal microbiota from curcumin-treated HFD-fed mice upregulated the expression and serum level of FGF15 in recipient mice (Fig. 6G–I). Taken together, these findings indicated that curcumin upregulated FGF15 expression through gut microbiota modulation, implying that curcumin at least partly increased insulin sensitivity in HFD-fed mice through FGF15 upregulation.

## Discussion

Obesity is one of the risk factors of insulin resistance, and curcumin can mitigate HFD-induced obesity in mice [15, 20]. In order to rule out the possibility that curcumin may increase insulin sensitivity via reducing HFD-induced obesity in mice, thereby interfering with experimental results, we chose to measure the glucose and lipid metabolic phenotypes of the mice before a significant divergence in body weight could be detected between the two groups. Consistent with previous studies completed by other researchers and our phenotypic data, curcumin significantly upregulated the insulin-stimulated increase in Akt phosphorylation levels in predominant insulin-regulated peripheral tissues [23–25]. The abnormal enhancement of the hepatic gluconeogenesis and lipid synthesis both caused by insulin resistance are contributed to hyperglycemia and hepatosteatosis in patients with type 2 diabetes, respectively [22, 26, 27]. Curcumin not only decreased the mRNA expression levels of *Pck1* and *G6pc* which encode two key enzymes in the hepatic gluconeogenesis, but also reduced the expression of genes involved in hepatic DNL, including *Srebf1*, *Fasn*, *Acaca* and *Scd1*, thus further confirming that curcumin could improve insulin sensitivity in HFD-fed mice.

Several studies have shown that curcumin can modulate gut microbiota composition, while the poor oral bioavailability of curcumin has implied that its beneficial effects are exerted through gut microbiota modulation [17–20, 28, 29]. Therefore, measurements on the effects of curcumin on glucose and lipid metabolism were repeated in HFD-fed mice after their endogenous gut microbiota were depleted, providing a clue that the effects of curcumin on improving HFD-induced insulin resistance were dependent on gut microbiota. Following that, FMT was performed, and glucose and lipid metabolic phenotypes were measured in those recipient mice. Our findings indicated that curcumin-restructured fecal microbiota produced similar effects to curcumin, while also confirming that curcumin improved insulin sensitivity through gut microbiota modulation in HFD-fed mice. To the best of our knowledge, this is the first time that gut microbiota depletion and FMT experiments have been employed to testify whether the effects of curcumin on increasing insulin sensitivity are dependent on its modulation of gut microbiota in HFD-fed mice.

Although gut microbiota are involved in the regulation of glucose and lipid metabolism, the regulatory mechanisms remain to be elucidated. As a gut-derived hormone regulated by gut microbiota, FGF15 can inhibit the expression of two key enzymes in the hepatic gluconeogenesis, glucose-6-phosphatase and phosphoenolpyruvate carboxykinase, causing *Fgf15*-knockout mice to exhibit diminished glucose tolerance [30–33]. Furthermore, overexpression of the *Fgf15* human ortholog, *FGF19*, can mitigate HFD-induced glucose intolerance and insulin resistance in mice, which is similar to the ameliorative effects of curcumin on HFD-induced GLMDs in mice [31, 32]. Thus, we speculated that curcumin may affect FGF15 expression through gut microbiota modulation to promote insulin sensitivity in HFD-fed mice. To verify this hypothesis, we measured the ileal expression and serum level of FGF15 in HFD-fed mice before and after gut microbiota depletion and recipient mice from FMT experiments. These findings indicated that the upregulation of FGF15 by curcumin was dependent on gut microbiota, suggesting a mechanism by which curcumin increased insulin sensitivity in HFD-fed mice through gut microbiota. However, in the current study, we are unable to rule out the possibility that curcumin may ameliorate HFD-induced insulin resistance and GLMDs through other downstream effectors of gut microbiota. Therefore, *Fgf15*^−/−^ mice should be introduced in the future study to investigate whether FGF15 mediates the effects of curcumin on improving insulin sensitivity in HFD-fed mice.

## Conclusions

This study demonstrated that curcumin ameliorated HFD-induced GLMDs and increased insulin sensitivity, and verified that the beneficial effects of curcumin required its modulation on gut microbiota. Moreover, we found that curcumin upregulated the expression of the gut-derived hormone FGF15, and this upregulation was also dependent on gut microbiota. Our findings suggested that curcumin at least partly exert its effects on increasing insulin sensitivity via FGF15 upregulation in HFD-fed mice. This study provided a theoretical basis for the clinical application of curcumin in the treatment of metabolic syndrome and type 2 diabetes. In addition, it is hoped that our findings will provide novel ideas on nutritional manipulations of gut microbiota for the treatment of metabolic diseases.

## Supplementary Information

**Additional file 1: Fig. S1.** The average body weight of curcumin- and vehicle-treated mice before and after fed with HFD for 4 weeks (n = 8/group). **Fig. S2.** The average liver weight of curcumin- and vehicle-treated mice after fed with HFD for 4 weeks (n = 8/group). **Fig. S3.** The average body weight gain of curcumin- and vehicle-treated HFD-fed mice during endogenous gut microbiota depletion (n = 8/group). **Fig. S4.** The average body weight of endogenous gut microbiota-depleted HFD-fed mice before and after colonized with the microbiota harvested from curcumin- and vehicle-treated HFD-fed mice for 4 weeks (n = 8/group).

## Data Availability

The data and material used to support the findings of this study are available from the corresponding author upon request.

## Abbreviations

HFD: High-fat diet
TGs: Triglycerides
FMT: Fecal microbiota transplantation
PCR: Polymerase chain reaction
ELISA: Enzyme-linked immunosorbent assay
DNL: De novo lipogenesis
FGF15: Fibroblast growth factor 15
GLMDs: Glucose and lipid metabolic disorders
FXR: Farnesoid X receptor
SPF: Specific pathogen-free
BCA: Bicinchoninic acid
Rps18: Ribosomal protein S18
Pck1: Phosphoenolpyrivate carboxykinase 1
G6pc: Glucose-6-phosphatase, catalytic
Srebf1: Sterol regulatory element binding transcription factor 1
Fasn: Fatty acid synthase
Acaca: Acetyl-CoA carboxylase alpha
Scd1: Stearoyl-coenzyme A desaturase 1
GTT: Glucose tolerance test
ITT: Insulin tolerance test
PTT: Pyruvate tolerance test
Akt: Protein kinase B
ANOVA: Analysis of variance
