# Supplementary material for: Curcumin improves insulin sensitivity in high-fat diet-fed mice through gut microbiota
Source: Nutr Metab (Lond). 2022 Nov 8;19:76. doi: 10.1186/s12986-022-00712-1 (PMC9644619; doi:10.1186/s12986-022-00712-1)
Supplement: Supplementary file 1 — Additional file 1: Fig. S1. The average body weight of curcumin- and vehicle-treated mice before and after fed with HFD for 4 weeks (n = 8/group). Fig. S2. The average liver weight of curcumin- and vehicle-treated mice after fed with HFD for 4 weeks (n = 8/group). Fig. S3. The average body weight gain of curcumin- and vehicle-treated HFD-fed mice during endogenous gut microbiota depletion (n = 8/group). Fig. S4. The average body weight of endogenous gut microbiota-depleted HFD-fed mice before and after colonized with the microbiota harvested from curcumin- and vehicle-treated HFD-fed mice for 4 weeks (n = 8/group). [file 12986_2022_712_MOESM1_ESM.docx]

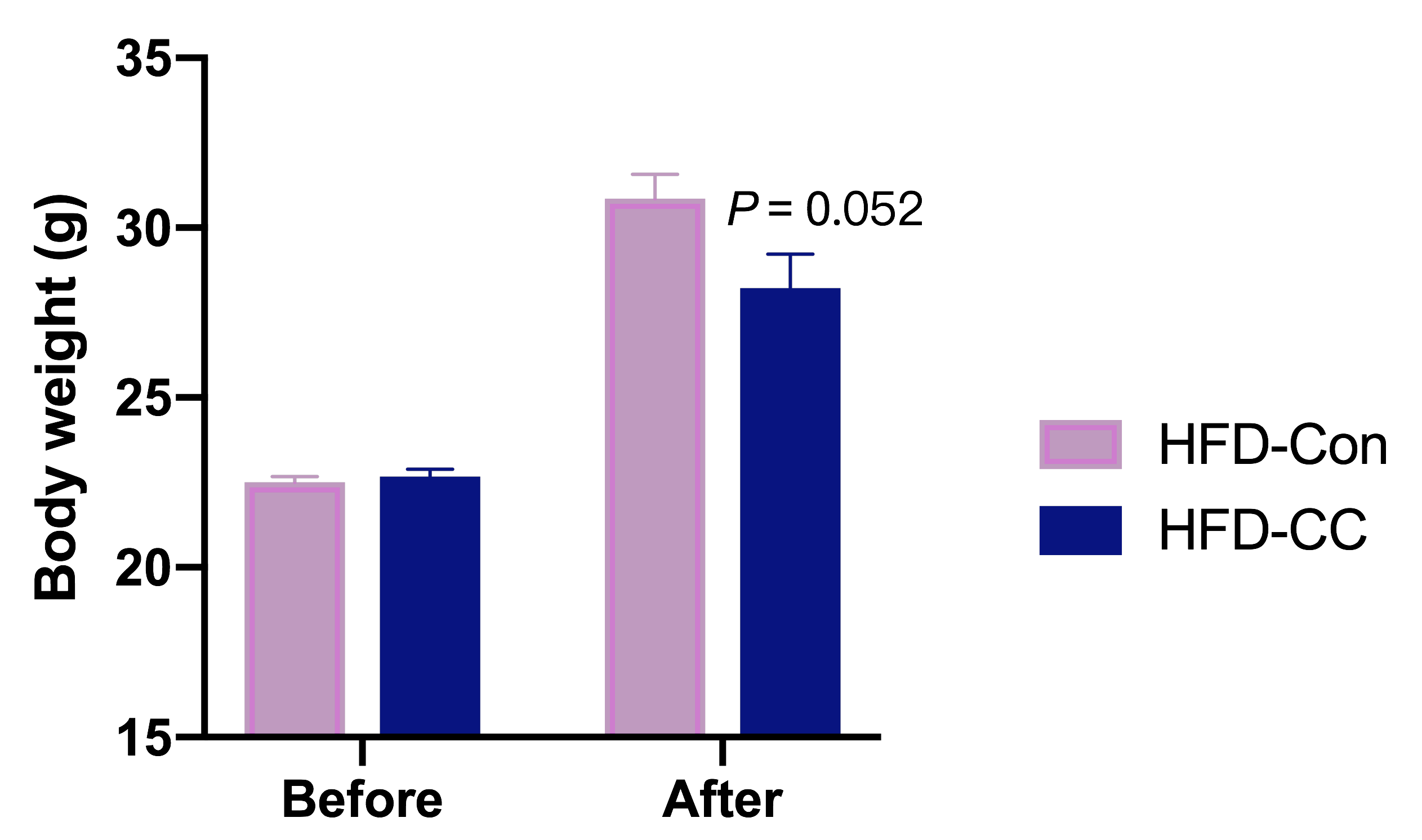


**Figure S1.** The average body weight of curcumin- and vehicle-treated mice before and after fed with HFD for 4 weeks (n = 8/group).


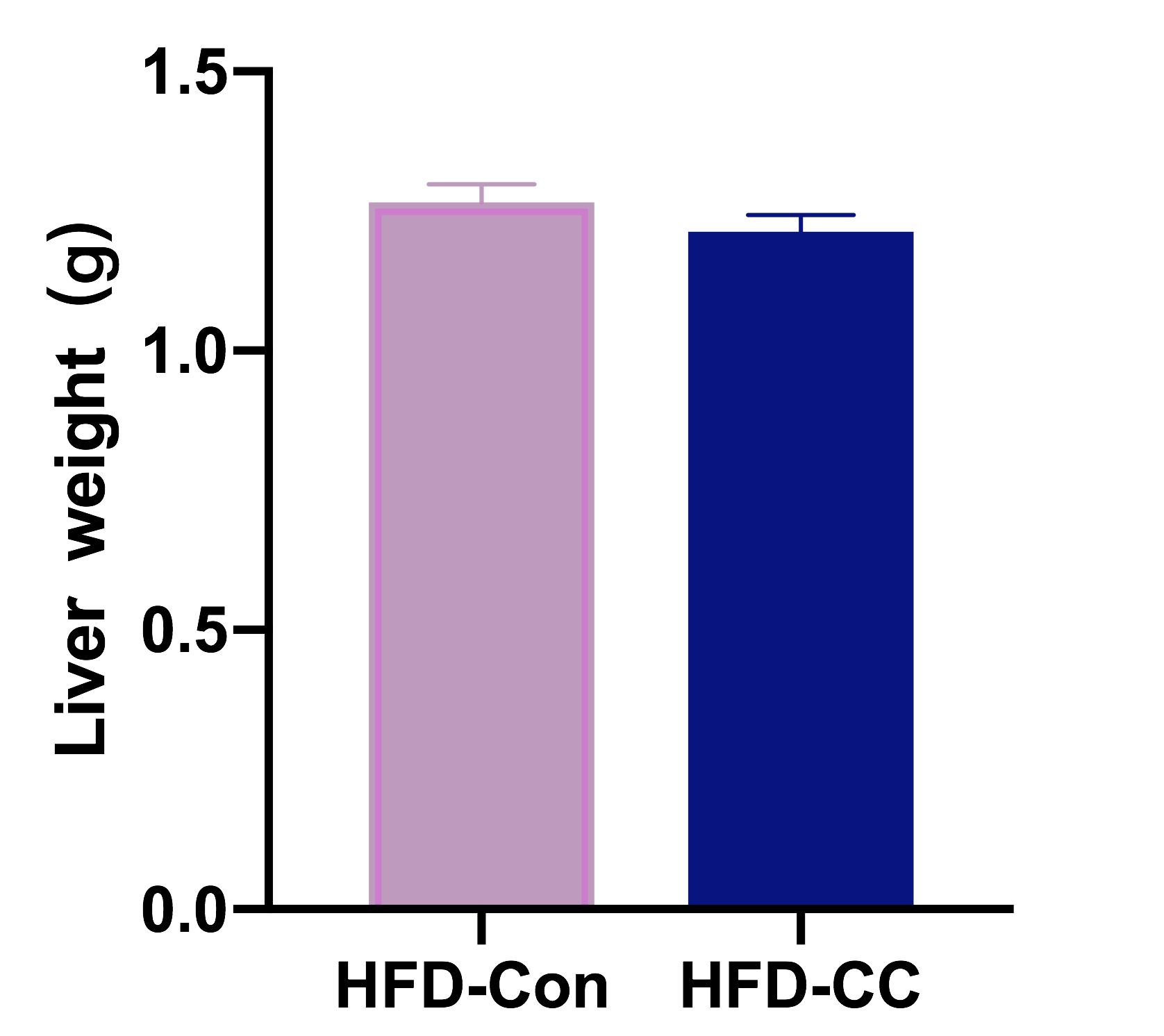


**Figure S2**. The average liver weight of curcumin- and vehicle-treated mice after fed with HFD for 4 weeks (n = 8/group).


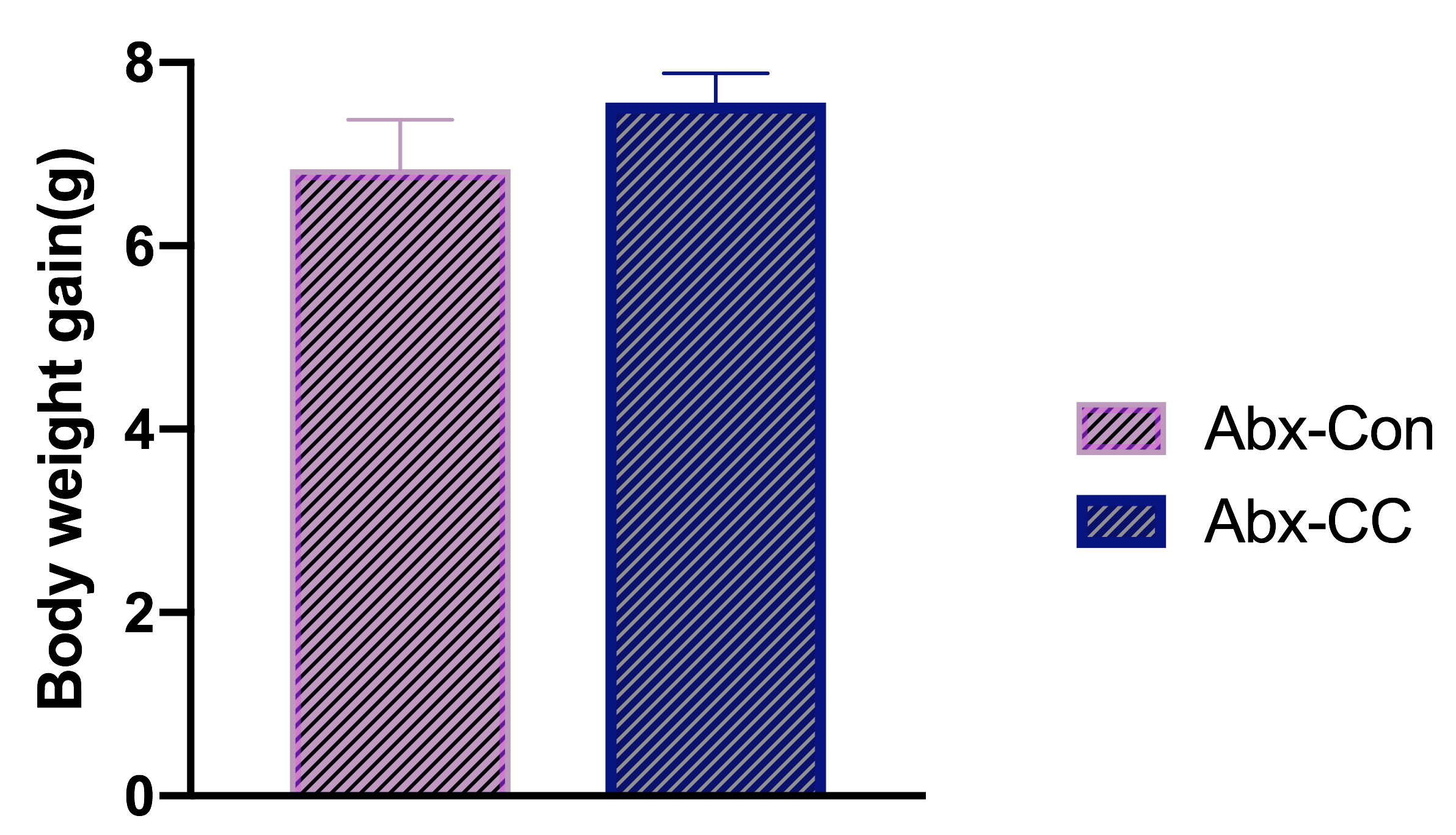


**Figure S3.** The average body weight gain of curcumin- and vehicle-treated HFD-fed mice during endogenous gut microbiota depletion (n = 8/group).


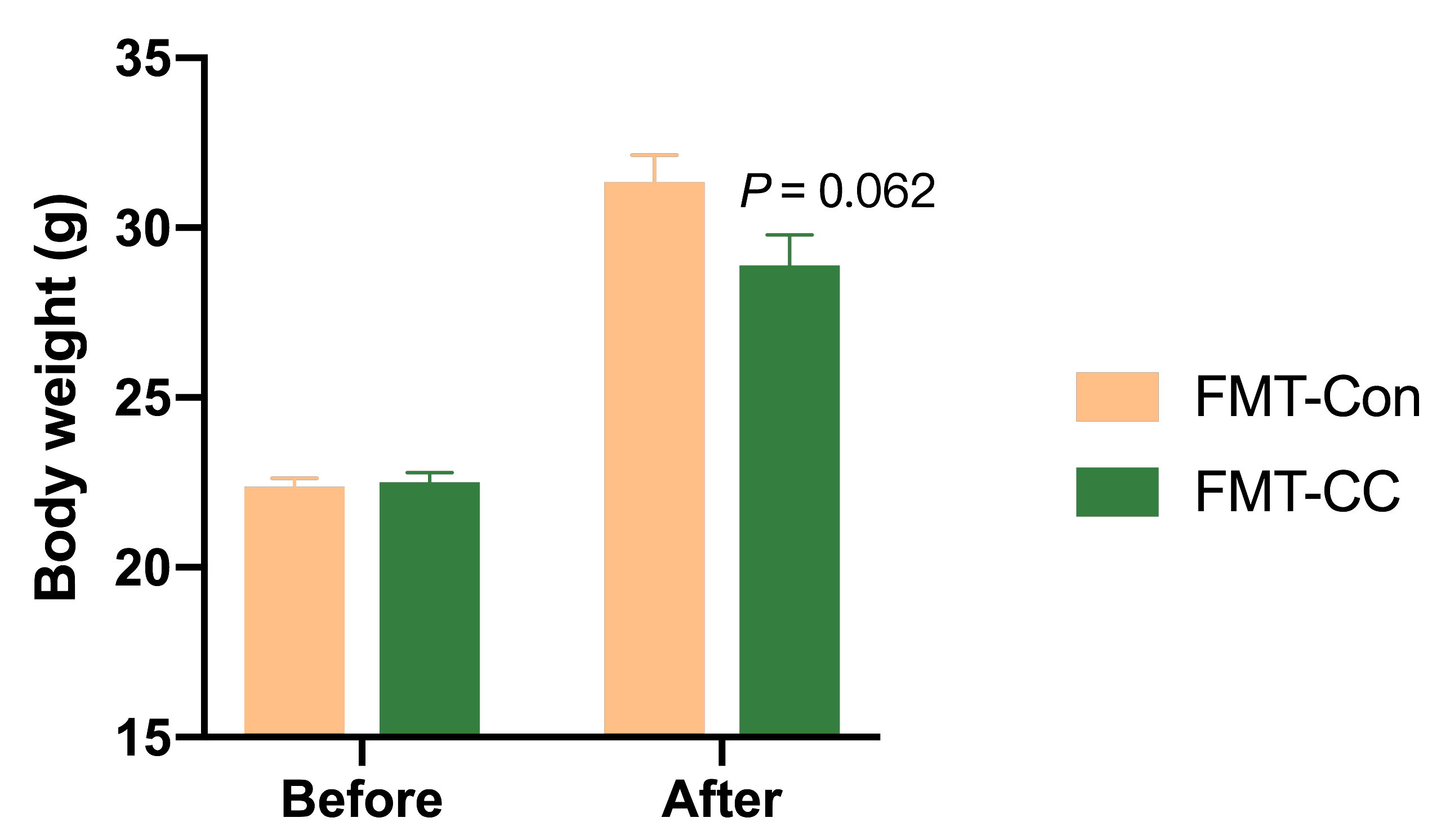


**Figure S4.** The average body weight of endogenous gut microbiota-depleted HFD-fed mice before and after colonized with the microbiota harvested from curcumin- and vehicle-treated HFD-fed mice for 4 weeks (n = 8/group).
